# The complete chloroplast genome sequence of medicinal plant: *Astragalus laxmannii* (Fabaceae)

**DOI:** 10.1080/23802359.2020.1829122

**Published:** 2020-11-03

**Authors:** Yi Liu, Yang Chen, Xi Fu

**Affiliations:** a Hospital of Chengdu University of Traditional Chinese Medicine, Chengdu, Sichuan, P. R. China; b Department of Nephropathy, Sichuan Integrative Medicine Hospital, Chengdu, Sichuan, P. R. China

**Keywords:** Chloroplast genome, phylogenetic analysis, Fabaceae, *Astragalus laxmannii*

## Abstract

*Astragalus laxmannii* is a traditional Chinese medicine. The complete chloroplast genome sequence is 122,844 bp in length, contains 110 complete genes, including 75 protein-coding genes (75 PCGs), 8 ribosomal RNA genes (4 rRNAs), and 30 tRNA genes (30 tRNAs). The overall GC content of cp DNA is 34.1%. Phylogenetic tree shows that *A. laxmannii* is a sister to *A. strictus*.

*Astragalus* species as one of the most important Qi tonifying adaptogenic herbs in traditional Chinese medicine (Yang et al., 2010; Zhong et al., 2012; Liu et al., 2017). It is valued for its ability to strengthen the primary energy of the body which we know as the immune system, as well as the metabolic, respiratory and eliminative functions (Liu et al., 2017). This fact is being increasingly substantiated by pharmacological studies showing that it can increase telomerase activity, and has antioxidant, anti-inflammatory, immuneregulatory, anticancer, antitumor, antioxidant, hypolipidemic, antihyperglycemic, hepatoprotective, expectorant, immunomodulatory activity, and diuretic effects (Anon 2003; Ma et al. 2011; Zhao et al. 2011). However, there are very few studies on the *A. laxmannii*, which greatly limit the development and utilization of *A. laxmannii*. So far, the chloroplast genome of *A. laxmannii* has not been reported. In this study, we assembled the complete chloroplast genome of *A. laxmannii*, hoping to lay a foundation for further research.

Fresh leaves of *A. laxmannii* were collected from Shapotou (Zhongwei, Ningxia, China; coordinates: 105ast genome of IN\\INdried with silica gel. The voucher specimen was stored in Sichuan University Herbarium with the accstion number of QTPLJQ13383120. Total genomic DNA was extracted with a modified CTAB method (Doyle and Doyle 1987) and a 350-bp library was constructed. This library was sequenced on the Illumina NovaSeq 6000 system with 150 bp paired-end reads. We obtained 10 million high quality pair-end reads for *A. laxmannii*, and after removing the adapters, the remained reads were used to assemble the complete chloroplast genome by NOVOPlasty (Dierckxsens et al. 2017). The complete chloroplasts genome sequence of *A. nakaianus* was used as a reference. Plann v1.1 (Huang and Cronk 2015) and Geneious v11.0.3 (Kearse et al. 2012) were used to annotate the chloroplasts genome and correct the annotation.

The total plastome length of *A. laxmannii* (MT786136) is 1,22,844 bp, exhibits a typical quadripartite structural organization, consisting of a large single copy (LSC) region of 66,532bp, two inverted repeat (IR) regions of 20,638 bp and a small single copy (SSC) region of 15,036 bp. The cp genome contains 110 complete genes, including 75 protein-coding genes (75 PCGs), 8 ribosomal RNA genes (4 rRNAs), and 30 tRNA genes (30 tRNAs). The overall GCcontent of cp DNA is 34.1%, the corresponding values of the LSC, SSC, and IR regions are 35.5%, 31.9%, and 43.5%.

In order to further clarify the phylogenetic position of *A. laxmannii*, plastome of nine representative *Astragalus* species were obtained from NCBI to reconstruct the plastome phylogeny, with *Oxytropis bicolor*as an outgroup. All the sequences were aligned using MAFFT v.7.313 (Katoh and Standley 2013) and maximum likelihood phylogenetic analyses were conducted using RAxML v.8.2.11 (Stamatakis 2014) under GTRCAT model with 500 bootstrap replicates. The phylogenetic tree shows that the species of *Astragalus*were divided into two subclades. *A. gummifer*, *A. mongholicus, A. nakaianus* and *A. membranaceus* clustered together. Remian species clustered in another clade, while *A. laxmannii* is a sister to *A. strictus*. (Figure 1).

**Figure 1.**
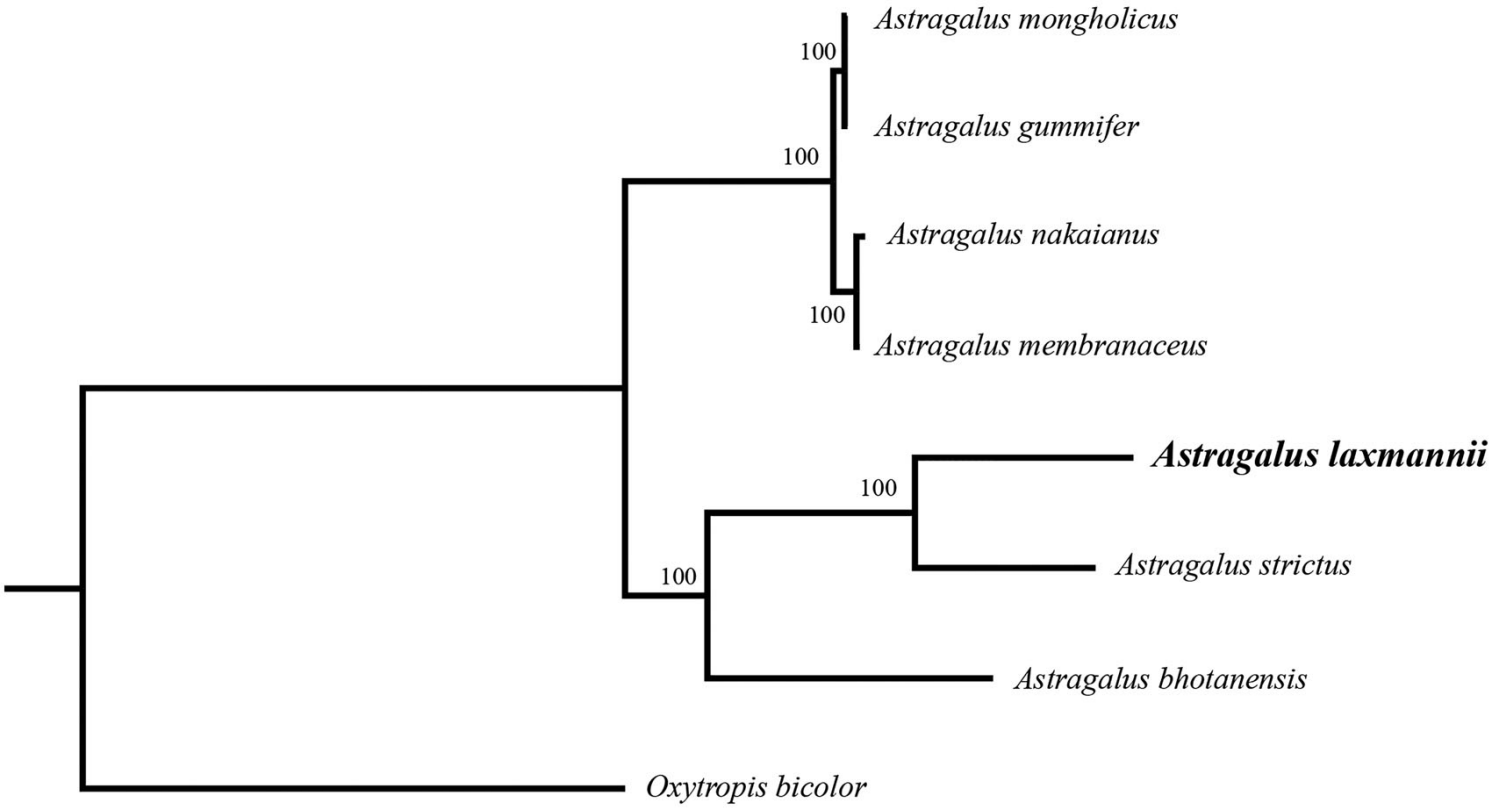
Phylogenetic relationships of *Astragalus* species using whole chloroplast genome. GenBank accession numbers: *Astragalus bhotanensis* (NC_047381), *Astragalus gummifer* (NC_047251), *Astragalus membranaceus* (KX255662), *Astragalus mongholicus* (NC_029828), *Astragalus nakaianus* (NC_028171), *Astragalus strictus* (MT120746), *Astragalus laxmannii* (MT786136), *Oxytropis bicolor* (NC_047482).

## Data Availability

The data that support the findings of this study are openly available in GenBank of NCBI at https://www.ncbi.nlm.nih.gov, reference number MT786136.
